# Modified Starch-Based Adhesives: A Review

**DOI:** 10.3390/polym14102023

**Published:** 2022-05-16

**Authors:** Jidapa Watcharakitti, Ei Ei Win, Jaturavit Nimnuan, Siwaporn Meejoo Smith

**Affiliations:** Center of Sustainable Energy and Green Materials and Department of Chemistry, Faculty of Science, Mahidol University, 999 Phuttamonthon Sai 4 Road, Salaya 73170, Thailand; bonusjidapa@gmail.com (J.W.); eieiwin.w96@gmail.com (E.E.W.); jaturavit.nim@gmail.com (J.N.)

**Keywords:** biopolymer, esterified starch, synthesis, adhesives

## Abstract

Consumer trends towards environmentally friendly products are driving plastics industries to investigate more benign alternatives to petroleum-based polymers. In the case of adhesives, one possibility to achieve sustainable production is to use non-toxic, low-cost starches as biodegradable raw materials for adhesive production. While native starch contains only hydroxyl groups and has limited scope, chemically modified starch shows superior water resistance properties for adhesive applications. Esterified starches, starches with ester substituents, can be feasibly produced and utilized to prepare bio-based adhesives with improved water resistance. Syntheses of esterified starch materials can involve esterification, transesterification, alkylation, acetylation, succinylation, or enzymatic reactions. The main focus of this review is on the production of esterified starches and their utilization in adhesive applications (for paper, plywood, wood composites, fiberboard, and particleboard). The latter part of this review discusses other processes (etherification, crosslinking, grafting, oxidation, or utilizing biobased coupling agents) to prepare modified starches that can be further applied in adhesive production. Further discussion on the characteristics of modified starch materials and required processing methods for adhesive production is also included.

## 1. Introduction

The global adhesives and sealants market is growing rapidly and is expected to be worth USD 85.8 billion by 2026 [1]. Industries such as those manufacturing packaging, plywood, and wood composites for furniture, electronic, aerospace and other technological products are fueling this expansion. Petrochemical feedstocks are typically used as the core raw materials to produce formaldehyde, urea, and polyurethanes, which are the key components of many widely used adhesives. However, environmental concerns about pollution from petroleum spills, global warming, and the depletion of fossil fuels have prompted researchers to look at using biodegradable ‘green’ materials as alternatives to petrochemical adhesive components [2].

Natural resources are the basis of ‘green’ raw materials for producing quality adhesives. Lignin, a renewable bio-based aromatic polymer derived from wood, and its derivatives have been utilized as substrates in polyurethanes, which are major adhesive constituents [3]. Functionalized cellulose (cellulose acetate) and castor oil have also been employed to produce bio-based polyurethane adhesives, which have suitable mechanical and adhesion properties for binding to wood surfaces [4]. Plant oil derived fatty acids were used as the basis for monomers which, once polymerized, form pressure-sensitive adhesives [5]. Phenol-formaldehyde resins, which are commonly found in wood adhesives, can be made more environmentally friendly by substitution of the toxic phenol for bio-derived, lower toxicity tannin [6].

Starch is one of the most abundant polysaccharides in nature, consisting of amylose and amylopectin in various ratios depending on the botanical source. Starch has been used extensively in food, medicine, and agricultural products. Due to its low cost, renewable nature and biodegradable quality, it is attracting the attention of plastic industries as a potential building block for petroleum-free adhesives, especially for paper and wood surfaces. However, the hydrophilicity of native starch makes formulating waterproof adhesives from it challenging as the starch hydroxyl groups easily form hydrogen bonds with water [2]. The introduction of hydrophobic functional groups (e.g., esters) onto native starch chains can improve its water resistance, and this can be carried out using chemical, mechanochemical, or enzymatic methods. This work emphasizes the up-to-date starch modification processes that have been used for adhesive applications. The primary focus of this paper is a discussion of the processes used for producing esterified starches, and the properties of the bio-adhesives obtained from such components. Additional sections outline modified starches obtained from etherification, crosslinking, grafting, oxidation, or bio-based coupling agents. The relationship between the properties of modified starch (i.e., degree of substitution,) and the properties (e.g., adhesion bond strength, water resistance, anti-retrodegradation) and types (water-based, solvent-based, and hot melt adhesives) of the obtained adhesives are propose at the end of this paper.

## 2. Esterified Starch

**Figure 1:**
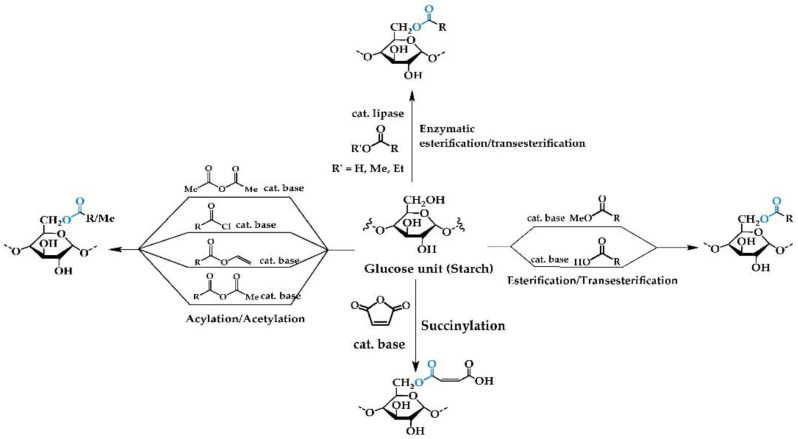
Chemical reactions for production of esterified starch.

Esterified starch is one of the important modified starches used in the adhesive and food industries. They are typically synthesized through esterification or transesterification reactions, in which the hydroxyl groups of starch are replaced with more bulky functional groups of free fatty acids or their derivatives (Figure 1).

### 2.1. Chemical Reactions

#### 2.1.1. Esterification/Transesterification

**Figure 2:**
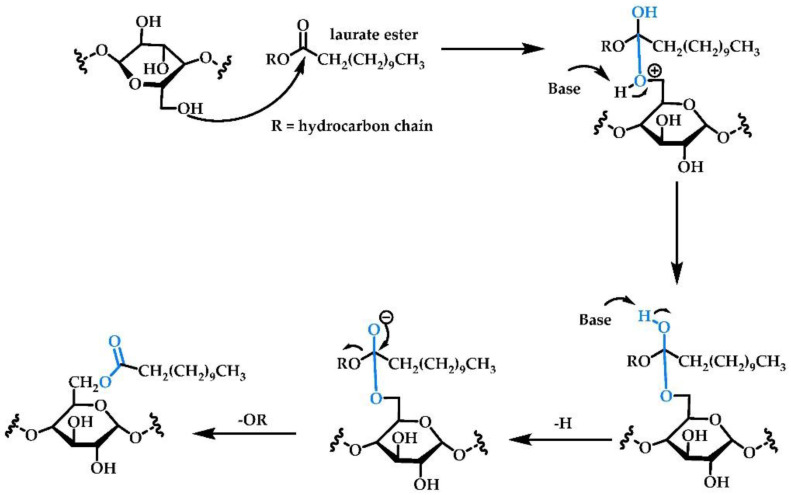
Transesterification of starch.

Esterification is one of the most powerful chemical synthesis strategies for starch modification. These reactions typically utilize alcohols and carboxylic acids in the presence of basic catalysts such as potassium carbonate, sodium hydroxide, and sodium hydrogen phosphate [7], or acid catalysts such as *p*-toluene sulfuric acid, hydrochloric acid, and sulfuric acid. The mechanism of esterification depends on the mode of catalysis, although studies by Sakamura et al. using isotopically labelled (^18^O) alcohol unequivically show that in both cases the ^18^O label is incorporated into the product at the end of the process, indicating bonding of this atom to the carbonyl carbon [8]. The mechanism of transesterification is similar but in this case involves the reaction of an alcohol with an ester, with the end result being loss of an alcohol in conjuction with formation of the esterified starch (Figure 2).

Examples of esterified starch have been reported by many research groups, with variations in ester carbon chain length. Ratnawati et al. [9] utilized glacial acetic acid to modify “Gadung flour” starch, which involved appending acetyl groups onto the saccharide unit in the presence of base catalyst (NaOH). This process afforded a product having a degree of substitution (the moles of substituents per mole of anhydroglucose unit) between 0.16 and 0.20. Under similar conditions starch extracted from *Colocasia esculenta*, a perennial, tropical plant, reacts with acetic acid, providing a product with higher degree of substitution (0.25–0.65) [10]. Starch citrates, obtained from the esterification of starch with citric acid, have been intensively studied for use in adhesives and food additives due to their antibiotic activity [11]. Moreover, the structure of starch citrates provides for cross-linking to be built into adhesives containing these bio-based polymers [12]. By employing transesterification, isopropyl myristate was reacted with cassava starch under conditions of acid catalysis. In this case the degree of substitution varied between 0.2 and 0.4, depending on reaction time [13]. As shown in the last example, esterification and transesterification strategies can be also used to produce starch modified with long hydrocarbon chains [14] for use in adhesives. However, such reactions can show limitations arising from steric interactions between the starch chain and the long hydrocarbon chain starting material, which results in low degrees of substitution. Enzymatic reactions to afford esterified starch under environmentally friendly mild conditions have also been reported [7,15]. Microwave induced esterification was employed to produce stearate starches with improved thermal stability in comparison to native starch [16].

#### 2.1.2. Acylation/Acetylation/Alkylation

Esterified starches can also be synthesized using acylation or acetylation reactions. In these cases, acylation involves appending the –C(O)R functional group to starch. Acetylation is a subset of acylation, which focuses only on the –C(O)CH_3_ group [17,18]. Compared to the previous section, acylation/acetylation involves the use of highly active acid chloride or anhydride starting materials, instead of carboxylic acids. Acid chlorides, due to their high relative reactivity [19,20], typically promote a higher degree of substitution than possible for carboxylic acids, and the R group on the acid chloride can be varied from short to long depending on application. Acid anhydrides can be employed in a similar manner [21,22]. However, these reactions have disadvantages in that these reagents are often more expensive and harder to handle than the corresponding carboxylic acids. In addition, the physicochemical properties of acetylated starch may also be affected by the acetylation reaction conditions, e.g., temperatures and pressure [23].

Starch modification through alkylation involves the formation of ether bonds in the product. Two common strategies for this utilize the reaction between starch and an alkyl halide [24,25], or epoxide ring-opening [26,27]. As an example of the former, etherified starch was synthesized through the reaction between allyl chloride and starch in the presence of pyridine. This etherified starch was used in a hydrogel application by reacting with methacrylic acid as a crosslinker [24]. The synthetic method of esterified starch is a major effect to determine the degree of substitution (being discussed in the following section). The comparison of the chemical reactions between esterification and acylation was studied. The acylation reactions promote the high degree of substitution product, but the esterification reactions do not give the high degree of substitution of esterified starch [28]. The reason behind the degree of substitution of product depends on the thermodynamic effect, reactivity of electrophile, and properties of leaving group. Moreover, the catalyst properties play an important role on the degree of substitution [29].

#### 2.1.3. Succinylation

Succinylation refers to the appending of side chains to starch by the reaction with succinic anhydride. Succinic anhydride is an electrophile, which undergoes nucleophilic attack by alcohol functionalities on the starch backbone. The esterified starch product has carboxylic acid functionalities at the terminus of each chain, and this allows the product to be incorporated into the adhesive directly or be further modified or functionalized prior to use. While succinylation results in a high degree of substitution [30,31] the limitation of this reaction is the cost of succinic anhydride. Additionally, the succinylation products have a carboxylic group at the terminal chain that could further promote cross-linking reactions between starch chains [32].

#### 2.1.4. Enzymatic Reactions with Free/Immobilized Lipase

Esterification of starch with fatty acids can be performed using enzyme catalysis, with lipases playing the key role. The use of enzymes has the advantage of not requiring extreme conditions and expensive starting materials, however the degree of substitution is often low [2,15,33] with immobilized lipase on magnetic microparticles providing a higher degree of substitution in the esterified starch than free lipase [34]. Horchani and coworkers also reported lipase immobilized calcium carbonate [35] as a catalyst in the esterification reactions between oleic acid and Maize starch. The yields of modified starch and their degree of substitution are varied, significantly depending on reaction conditions (temperature, pH, and lipase enzyme origin) [36]. Obtaining lipase from either plant of microorganisms requires multi-step isolation, purification and characterization of lipase to ensure its high purity and enzamic activity [35,37]. Additional limiations of enzymes such as low thermostability, pH sensitivity, narrow substrate scope, and low reaction yield in non-aqueous solvents [38], may result in restrictions in starch modification processes.

#### 2.1.5. Mechanochemical Processes

These processes involve reactions taking place without the addition of solvent and utilize mechanical activation, such as ball milling. In the case of starch esterification, ball milling allows for intimate mixing of starch and the esterification agent, giving good yields of product [39]. This processing also disrupts the structure and morphology of the starch, and results in changes in physicochemical properties, compared to those of native starch, such as decreased cold-water solubility, transparency, and emulsion stabilization, along with increased gelatinization temperature and viscosity [32,40,41].

### 2.2. Properties of Starches

The ratio of amylose to amylopectin in starch is dependent on its botanical source, as shown in Table 1. Amylose is a linear polymer of glucopyranose units linked through α-1,4-glycosidic linkages, in contrast to amylopectin which is a branched polymer. Starch crystallinity depends on the proportion of amylopectin, as amylose is typically amorphous. Differences in amylose content and granule organization influence the gelatinization of the starch, with starch granules containing higher proportions of amylose being easily gelatinized by heating as the chains are only weakly associated through hydrogen bonding [42].

#### 2.2.1. Degree of Substitution

The degree of substitution (DS) in esterified starch is an important factor dictating the physical properties of the material. It may be determined using ^1^H-NMR spectroscopy or through an acid–base titration process. Examples of the DS of selected modification are listed in Table 2.

##### Determination of DS *via* Titration

The degree of substitution (DS) is often determined by acid–base titration due to its convenience [45]. This protocol involves dissolving the esterified starch in 0.5 M NaOH solution under ambient conditions, followed by incubation with shaking (300 rpm, 30 °C) for 4 h. After that, the reaction is titrated against 0.1 M HCl solution, and the degree of substitution (DS) is determined using Equation (1).

DS = (162[HCl](V_0_ − V_1_))/(1000 × sample mass (g)) (1)

In Equation (1), [HCl] is the concentration of the HCl solution, while V_0_ stands for the initial volume of hydrochloric acid (V_0_ > 0), V_1_ stands for the final volume of hydrochloric acid.

##### Determination of DS *via* ^1^H-NMR Spectroscopy

This method requires identification of specific protons related to the ester units in the product, and integration of their relative areas in ^1^H NMR spectra. The DS can then be calculated using Equations (2) and (3) [7]. This method requires acetyl group substitution onto the starch unit for DS to be reliably determined. In Equation (3) I is the integration of the ^1^H NMR peaks corresponding to acetate or starch H-atoms.

DS_fatty ester_ = 3 − DS_acetate_ (2)

DS_acetate_ = I_H-acetate_/I_H-starch_ (3)

#### 2.2.2. Morphology of Native and Modified Starches

The morphology of starch granules (native and modified) can be studied using scanning electron microscopy (SEM), and this technique provides information about the granule shape, and surface features. Starch granules are the vehicle of carbohydrate storage in plant cells, and the morphology of these depends on the biochemistry of the chloroplast/amyloplasts and the physiology of the plant. Normally, native starch granules are elliptical, have a smooth surface, and range from 1 to 100 μm in size [46]. Modification can result in the granules becoming irregular in shape with pronounced surface roughness, especially at high degrees of substitution (DS) [46,47,48].

#### 2.2.3. Viscosity of Starch Pastes

In addition to the source of starch and conditions used to make the paste, functionalization of the starch can also markedly affect the viscosity of the paste. A study on the paste viscosity of OSA-esterified starch [32,49] indicated that paste obtained from this material was higher than that of native starch, as a consequence of the bulky OSA functional groups. Acylation of starch gives rise to steric interactions between starch chains, and this influences both hydrophilicity and the degree of hydrogen bonding, and thus viscosity. The viscosities of esterified starches generally decrease as the molecular weight of the ester group increases [50].

#### 2.2.4. Solubility/Hydrophobicity

Appending larger functional groups onto the starch units results in increased hydrophobicity and lower water solubility. On the other hand, solubility in lower polarity solvents increases [2] and this impacts the potential application of these starch derivatives [47].

#### 2.2.5. Thermal Properties of Starches

The thermal properties of starch are dependent on the amylose content or amylose/amylopectin ratio [51,52], and are also affected by functionalization. While esterified starches having low degrees of substitution (DS) do not see dramatic changes in onset temperature relative to native starch, starches having higher DS values show a decrease in gelatinization temperature [53]. Furthermore, the glass transition temperature (T_g_), the temperature at which a glassy state of amorphous materials converts into a rubbery state, is affected by structure. In the case of functionalized starch, steric hindrance between starch chains increases as the functional groups become larger, resulting in higher T_g_. On the other hand, the extension of free volume of chain mobility can reduce T_g_ [53]. 

The improved thermal stability of starch can be reported based on their gelatinization, retrogradation, and glass temperature. In Table 2, the properties of starch esters derived from different processes (acylation, acetylation, esterification, transesterification, enzymatic reactions) are highlighted. As indicated, the reagents employed can vastly impact the properties and degree of substitution (DS) in the esterified starch products. The starch esters can be categorized by their DS values into three levels: low (0.01–0.2) which are water soluble, and intermediate (0.2–1.5) and high (1.5–3) which are thermoplastic but more hydrophobic [51]. Oil feedstocks typically afford starch esters having lower DS values than those utilizing very reactive electrophilic substrates such as acid chlorides [47,72] and acid anhydrides [17,18]. In addition, the use of vinyl laureate and vinyl stearate afford broad DS ranges in the presence of base catalysts (Na_2_HPO_4_, K_2_CO_3_, and sodium acetate), with base strength correlating with DS [62]. When considering the same substrate and conditions, stronger base catalysts result in higher DS values (up to 2.96) [7]. 

Other works [50,58] also found that the transesterification of starch with fatty acid vinyl ester provides a high DS starch ester products compared to methyl laurate ester [33,40] in both catalyst-free reaction and in the presence of a catalyst. For enzymatic reactions, free enzymatic catalysts using native starch provided starch esters with lower DS values [62] than those produced using immobilized lipase as catalyst [35]. The immobilized catalyst used was composed of lipase and calcium oxide, with the synergy between the two components believed important for enhanced activity [35]. The type of acyl donor also influences the viscosity of the enzymatically modified starches, as the viscosity of the palmitic acid modified starch was found higher than that of the lauric acid modified one [62,63]. Many esterified starches reported to date show higher thermal stability or higher decomposition temperatures than native starch, and greater degrees of hydrophobicity.

### 2.3. Adhesives Derived from Esterified Starch

Starch-based adhesives are widely used in the production of plywood [30,73] and particleboard [74,75]. Cassava starch esterified using dodecyl succinic anhydride (DDSA) as a reactant [30] and crosslinked with polymethylene polyphenyl polyisocyanate (PAPI) results in a formaldehyde-free industrial plywood adhesive with excellent viscosity, bonding strength, and water resistance properties [30]. Natural corn starch was converted to high amylose corn starch (HACS) by solvent exchange with ethanol and toluene prior to esterification with propionic anhydride in the presence of catalytic 4-dimethylaminopyridine (DMAP). The DS of these starch propionates was in the range 0.38–2.54, with higher ratios of propionic anhydride which enhanced the adhesion strength relatively to native starch. These modified starches were mixed with glycerol and polyvinyl alcohol and hot-pressed to form hot melt adhesives on Al plates. It was found that these covered a greater surface area and exhibited higher tensile strength than adhesives formed analogously from unmodified starch [76]. As mentioned in earlier sections esterified starch from chemical modifications show enhanced hydrophobicity and water resistance, with adhesives formed from this exhibiting improved bonding strength under both dry and wet conditions relative to those from natural starch [77] (Table 3).

**Figure 3:**
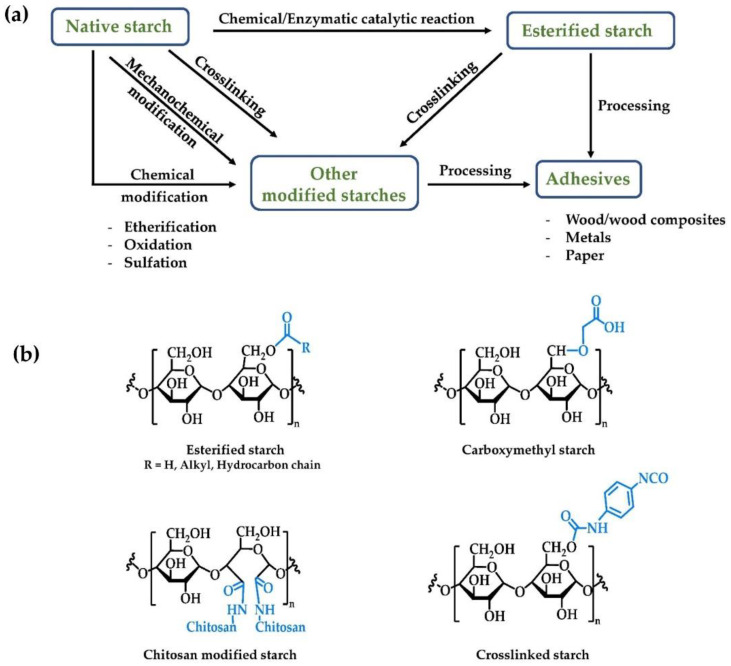
(**a**) Selected examples of starch modification processes being used for adhesive applications and (**b**) examples of the structure of modified starches. Selected examples of starch modification reactions, with given details in Table 4.

Figure 3a illustrates general starch modification processes that have been used in production of starch-based adhesives, while the structure of selected modified starches is given in Figure 3b. Apart from esterified starches, biobased adhesives can be obtained by using other chemically modified starches such as carboxymethyl-, chitosan modified-, and crosslinked starches as raw materials. Details some starch medication methods are given in Table 4 with additional discussion as follows.

## 3. Other Modified Starches for Adhesive Applications

The application of native starches in adhesives is limited due to their relatively poor shear strength, low thermal stability, and hydrophilicity [84]. Chemical modification is a successful strategy for changing the structure and properties of biopolymers to fit new applications [84] with esterification of starches discussed in the previous section being a prime example. In addition to esterification other modifications of starches can include etherification, oxidation, crosslinking, and grafted copolymerization, as shown in Figure 3 and Table 4.

Etherification can be used to modify the water resistance properties of starch through the introduction of lipophilic functional groups. This is generally accomplished by treating starch with epoxides, such as propylene or ethylene oxides. These epoxides are highly reactive, with the starch OH groups attacking the ring which results in cleavage of the C-O bonds of the epoxide, affording diol products. The lipophilic alkyl groups produced are subsequently linked to the starch chains, thus increasing the hydrophobicity of the starch and improving its water resistance. [85]. Hydroxypropylation, hydroxyethylation, and carboxymethylation are three such etherification procedures for preparation of starch ethers [75]. Carboxymethyl starch (CMS) is widely employed in adhesive manufacturing due to its favorable viscosity and stability [86]. CMS adhesives can be synthesized by wet methods using aqueous media, or dry processes without the use of solvent [87].

Crosslinking, the process of forming nonpolar covalent bonds between the hydroxyl groups of starch, is also a strategy for improving the utility of starches in adhesives. Crosslinked starch exhibits superior mechanical (tensile strength), thermal, and water stability compared to native starch. [88,89,90]. Furthermore, crosslinking acts to inhibit acid degradation, resulting in sustained viscosity in acidic media. A variety of crosslinking agents can be employed in starch modification [91]. Among these citric acid, sodium trimetaphosphate (STMP), sodium tripolyphosphate (STPP), epichlorohydrin (ECH), and phosphorus oxychloride (POCl_3_) are the most utilized [16]. Detduangchan et al. studied the effects of chemically crosslinking rice starch films using ECH, STMP, and STMP/STPP mixtures. After crosslinking, the tensile strength increased from 5.01 MPa (native starch) to 8.23 MPa, and the water permeability decreased, implying enhanced adhesive water resistance [88].

Another convenient method to modify starch is by grafting synthetic monomers or polymers with desirable properties onto the natural starch backbone. In these instances, it is desirable if the crystallinity and biodegradability properties of the starch are unchanged. Grafting typically occurs at the Cl–C2 end groups, and C2–C3 glycol groups on the starch glucose units. Graft copolymers produced from addition of vinyl- or other monomeric acrylates show improved water resistance and shear strength over natural starch [92]. The use of two co-monomers (vinyl acetate and butyl acrylate) resulted in starch adhesives exhibiting improved shear strength, and requiring lower film-forming temperatures, than those of natural starch [48,93]. Such adhesives retain the biodegradable profile of natural starch while being less hydrophilic, exhibit greater tensile strengths, and maintain a better appearance over time [94].

The unique properties of nanoparticles such as their small size, high surface energy, and the ability to functionalize their surfaces render them attractive for the development of high-performance composite materials. Nano-TiO_2_, nano-SiO_2_, and montmorillonite (MMT) nanoparticles have been used as crosslinking or grafting agents to improve the characteristics of biopolymer-based adhesives in recent years [95,96]. Significant improvements in water resistance and bonding strength were found when nanoTiO_2_, [97,98] saline coupling agents [95], vinyl acetate [93], acrylate [73,93] and polyamide [99,100] were used as crosslinking or grafting agents with the starch for adhesive purposes.

Natural polymers derived from biomass (lignin, cellulose, hemicellulose), and chitosan have the potential to be used as bridging agents in adhesive formulations. These may be useful in systems containing inorganic materials and organic components such as starch, and their use can improve the adhesive properties of the formulation [100,101,102]. Blending starch with hydrophobic biopolymers (such as lignin) could be a strategy for improving the water-resistance of the adhesive and altering its mechanical properties.

Prior to crosslinking or grafting, starch is often pretreated using acid hydrolysis or oxidation [100]. Acid hydrolysis involves breaking the native starch polymer into shorter chained polysaccharides containing amylose and amylopectin subunits through treatment with acid at low temperature. This results in active polysaccharides subunits which react readily with crosslinking agents, affording modified starch networks having superior strength and reduced retrogradation (starch recrystallization upon cooling) [85]. Acid modified starch adhesives have been found to be more water resistant and exhibit superior bonding strengths [41].

Unlike acid hydrolysis, oxidation causes partial depolymerization of native starch, creating free carboxylic or aldehyde groups from the oxidation of primary and secondary hydroxyl groups. The oxidation of native starch involves loss of crystallinity due to a reduction in the number of network hydrogen bonds. The remaining carboxyl and hydroxyl groups subsequently react with other polymers or monomers to form covalent bonds, which result in changes in adhesive properties and starch stability. While periodate, chromic acid, potassium permanganate, nitrogen dioxide, and sodium hypochlorite are often employed, sodium hypochlorite is the widely used oxidant in industry as it results in adhesives having increased tensile strengths [85]. Moreno et al. [103] prepared biodegradable active films from corn starch oxidized using sodium periodate. These films showed enhanced tensile strength and oxygen and water vapor barrier capacities, and reduced migration to acid media when compared with native starch films. Compared to oxidation alone, starch modification by oxidation and subsequent crosslinking can provide adhesives exhibiting higher shear strengths and lower tendency for retrogradation to occur [104].

In addition to the results of synthetic studies, the properties of native starches may be predicted computationally. Computational and mathematical modeling at the molecular level may allow for selection and optimization of reaction conditions for producing adhesives tailored to specific applications. For instance, sulfation of potato starch using formic acid/urea is a simple and environmentally friendly method to obtain starch sulfates [105]. Furthermore, sulfation of starches by using sulfamic acid and urea or other sulfating agents (e.g., H_2_SO_4_, HSO_3_CL, and SO_3_) could result in the modified starches having biological activity (i.e., anticoagulant, antioxidant, antiviral and anti-inflammatory activity) [105]. Sulfated starches have attracted a great attention in the area of therapeutic agents against chronic diseases [106] rather than as adhesives’ raw materials. The feasibility of utilizing sulfated starches to produce adhesives should be further explored and examined.

## 4. Conclusions and Perspectives

Utilization of natural resources or bio-based materials as adhesive raw materials could help future societies to become less reliant on hazardous chemicals, volatile organic compounds, and finite petroleum-based chemicals [114]. Formaldehyde-free or biobased adhesives could promote safer working conditions in related industries [115,116]. Although improved water resistance in adhesives can be achieved using modified starches as raw materials instead of native starches, the effects of starch quality on the properties of the adhesives warrants further investigation. Quality variations in natural additives (e.g., lignin, cellulosic materials, chitosan) could also create difficulties in obtaining products of uniform composition and performance, and this may be a barrier to their uptake by industry. Despite these challenges, the potential of modified starches in bio-based adhesive manufacturing is clear. Moving towards environmentally sustainable adhesives requires scaling back the use, or abolition of, volatile organic solvents in adhesive formulations. Much research needs to be conducted on the selection and compatibility of ‘green’ solvents for use alongside adhesive components. Additionally, solvent-free adhesives (such as hot-melt adhesives) derived from insoluble modified starches should be also investigated further as safe, environmentally friendly alternatives.

## Figures and Tables

**Table 1:** Properties of native starch [43,44].

Type of Starch	Ratio of Amylose to Amylopectin	Gelatinization Temperature (°C) ^a^	% Solubility	% Crystallinity ^b^
Rice	30:70	68–77	11–18, 95 °C	38
Potato	18:82	58–68	82, 95 °C	23–53
Cassava	18:82	60–80	-	31–59
Wheat	20:80	58–64	1.55, 100 °C	43–48
Corn	28:72	62–72	22, 95 °C	22–28

Differential scanning calorimetry (a) and X-ray diffraction (b) were utilized to obtain the gelatinization temperature and %Crystallinity, respectively.

**Table 2:** Reactions affording starch esters and their degree of substitution.

Modification of Starch		Type of Starch	Degree of Substitution (DS)	Ref.
Reaction	Reagent–Catalyst			
Acylation	Carboxylic acid (butanoic, hexanoic, octanoic, palmitic acid)	Potato	2.52–3.00	[54]
Acetylation	Acetic anhydride-iodine	Corn	0.12–2.97	[17]
	Acetic anhydride-tartaric acid	Corn	0.06–1.54	[21]
	Acetic anhydride and glacial acetic acid	Potato	0.015–0.054	[55]
	Vinyl acetate	Amaranth grain	0.22	[56]
Esterification	Carboxylic acid imidazolides (C_8_, C_12_, C_16_)—methanolic KOCH_3_	Potato	1.76	[57]
	Lauric acid—K_2_CO_3_	Cassava	0.0148–0.0412	[39]
	Lauroyl chloride	Corn	0.45–2.92	[47]
	Lauric acid, palmitic acid, and stearic acid	Corn	0.053–0.100	[58]
	Stearyl chloride	Corn	0.25–1.58	[59]
	Lactic acid—stannous octoate	Corn	0.015–0.12	[60]
	Citric acid	Rice	0.015–0.064	[61]
Enzymatic esterification	Oleic acid—immobilized lipase	Maize	2.86	[35]
	Hydrolyzed recovered coconut oil (lauric acid) and palmitic acid—fungal lipase	Cassava	1.1 and 1.04	[62]
	Lauric acid-lipase in ionic liquid	Maize	0.048–0.171	[33]
	Carboxylic acid (acetic, lauric, and stearic acid)-lipase	Maize	0.016–0.513	[15]
	Recovered coconut oil-lipase	Maize	1.55	[63]
	Hydrolyzed rapeseed oil—immobilized fungal lipase	Potato	0.15–1.36	[64]
	Palmitic, lauric, decanoic acids—lipase in deep eutectic solvent	Native	0.07–0.19	[65]
Transesterification Transesterification	Olive oil or high oleic sunflower oil-TBD	Maize	1.29–1.33	[66]
	Fatty acid vinyl ester	Maize	1.40–1.73 and 2.20–2.63	[67]
	Vinyl laurate—Cs_2_CO_3_	Maize	1.75–2.44	[68]
	Methyl laurate—potassium laurate	Maize	0.08–0.62	[69]
	Vinyl laurate and vinyl stearate—Na_2_HPO_4_, K_2_CO_3_ and Na acetate	Corn	0.24–2.96	[7]
	Methyl laurate	Corn	0.2673–0.7034	[70]
	Fatty acid methyl ester	Sago	0.45	[71]

**Table 3:** Esterified starch derived adhesives. The properties are reported relatively to those of corresponding native starches.

Starch	Modification	Properties	Utilization	Ref.
Cassava	Esterification using dodecenyl succinic anhydride (DDSA) as reactant and polymethylene polyphenyl polyisocyanate (PAPI)	Improved bonding strength and water resistance of esterified starch adhesive	Adhesive film for plywood	[30]
Cassava	Grafted with olein monomer (vinyl acetate; VAc and butyl acrylate; BA as co-monomer)	Enhanced the storage stability of starch wood adhesive and glass transition temperature	Wood adhesive	[73]
Cassava	Grafted with different typrs of acrylic ester	Improved performance and shear strength under both dry and wet conditions	Wood adhesive	[48]
Cassava	Esterification with acid in bio-oil and grafted copolymerization with vinyl acetate and butyl acrylate	Lower viscosities, improved shelf life and mildew resistance of bio-adhesive	Wood adhesive	[78]
High amylose corn starch (HACS)	Esterification using propionic anhydride as agent and catalytic 4-dimethyl aminopyridine (DMAP)	Surface area of HACS was increased with improved stability, hydrophobicity, and adhesion strength	Hot melt adhesive bonded Al plate	[76]
Corn	Esterification using maleic anhydride	Improved shear strength and thermal stability	Wood adhesive	[79]
Corn	Grafted with vinyl acetate and crosslinked with *N*-methylol acrylamide	Improved water resistance and can be used in hot pressimg processes	Wood-based panel adhesive	[80]
Corn	Addition of sucrose fatty acid ester	Enhanced shear-thinning, solid-like behavior, and anti-retrodegradation of starch	Wood adhesive	[81]
Corn	Esterification with phthalic anhydride in DMF	Reduced viscosity and thermal decomposition	Adhesive	[82]
Waxy corn	Grafted with vinyl acetate	Increased shear strength under both dry and wet conditions	Wood adhesive	[77]
Potato	Transesterification with natural oil and combined with toluene 2,4-diisocyanate	Good resistance to cold and hot water, moderate resistance to acid and weak resistance to akali	Polyurethane (PU) adhesive for wood	[83]

**Table 4:** The preparation of modified starch and adhesive application from different starch.

Reaction of Modified Starch	Starch	Properties	Utilization	Ref.
Oxidation and modification with chitosan	Corn	Improved dry and wet shear strength of plywood	An adhesive film for plywood	[102]
Oxidation using H_2_O_2_ and then crosslinking with B-pMDI and citric acid	Corn	Improved physical properties, mechanical properties, and water resistance	Medium density fiberboard	[107]
Oxidation using KMnO_4_, then crosslinking and copolymerization with polyamide and methyl methacrylate	Corn	Improved wet shear strength and water resistance	An adhesive for plywood	[108]
Oxidation using KMnO_4_, polycondensation reaction with urea and addition of nano-TiO_2_	Corn	The nano-TiO_2_ effectively improves dry shear strength and viscosity of the nano-TiO_2_-U-OSt adhesive.	An adhesive	[98]
Oxidation using H_2_O_2_ and crosslinking with polyamidoamine-epichlorohydrin (PAH)	Rice	Enhanced thermal stability, hydrophobicity, wet-cohesion, and adhesiveness	An adhesive for wood composites	[100]
Etherification with carboxymethyl and use of POCl_3_ as crosslinking agent	Wheat	The modified starch mixed with PVA improves solid content, heat and water resistance but decrease viscosity.	Adhesive for particleboard	[75]
Etherification with epichlorohydrin	Oil palm	Improved mechanical strength (modulus, elasticity, and internal bond), solid content and viscosity	Adhesive for particleboard	[109]
Graft copolymerization with glycidyl methacrylate (GMA) and crosslinking with sodium trimetaphosphate (STMP).	Cassava	Improved water resistance and bonding strength	An adhesive for plywood	[110]
Graft copolymerization with sodium dodecyl sulfate (SDS)	Micronized (MS)	Improved shear strength and decreased viscosity of micronized starch with increasing SDS contents	Wood adhesive	[111]
Graft copolymerization with lignin	Corn	Improved adhesive bond strength and moisture resistance, including extended shelf-life.	An adhesive for paper	[101]
Crosslinking with polyphenylene isocyanate (PAPI) with poly vinyl alcohol (PVOH) as a protective colloid	Cassava	Improved water resistance, shear strength, mobility and storage stability of starch adhesive	Wood adhesive	[112]
Crosslinking with lignin	Corn	Lignin improved the strength and water resistance of adhesive	An adhesive for cardboard application	[113]
